# Using liquid chromatography mass spectrometry (LC-MS) to assess the effect of age, high-fat diet, and rat strain on the liver metabolome

**DOI:** 10.1371/journal.pone.0235338

**Published:** 2020-07-01

**Authors:** Greg Boyce, Mohammad Shoeb, Vamsi Kodali, Terence Meighan, Jenny R. Roberts, Aaron Erdely, Michael Kashon, James M. Antonini

**Affiliations:** National Institute for Occupational Safety and Health, Morgantown, West Virginia, United States of America; Queen’s University Belfast, UNITED KINGDOM

## Abstract

The goal of this study was to use liquid chromatography mass spectrometry to assess metabolic changes of two different diets in three distinct rat strains. Sprague-Dawley, Fischer 344, and Brown-Norway male rats were maintained on a high-fat, or regular diet for 24 weeks. Liver tissue was collected at 4, 12, and 24 weeks to assess global small molecule metabolite changes using high resolution accurate mass spectrometry coupled to ultra-high-performance liquid chromatography. The results of the global metabolomics analysis revealed significant changes based on both age and diet within all three strains. Principal component analysis revealed that the influence of diet caused a greater variation in significantly changing metabolites than that of age for the Brown Norway and Fisher 344 strains, whereas diet had the greatest influence in the Sprague Dawley strain only at the 4-week time point. As expected, metabolites involved in lipid metabolism were changed in the animals maintained on a high fat diet compared to the regular diet. There were also significant changes observed in the concentration of Tri carboxylic acid cycle intermediates that were extracted from the liver of all three strains based on diet. The results of this study showed that a high fat diet caused significant liver and metabolic changes compared to a regular diet in multiple rat strains. The inbred Fisher 344 and Brown Norway rats were more metabolically sensitive to the diet changes than outbred Sprague Dawley strain. The study also showed that age, as was the case for Sprague Dawley, is an important variable to consider when assessing metabolic changes.

## Introduction

Obesity is increasing in many populations around the world and causes major health effects and exacerbation of disease [1, 2]. Currently over 30% of the world’s population is considered obese and that number is projected to grow [2]. There are many factors that contribute to obesity including genetic predisposition, physical activity, age, and diet. The modern Western diet consist of high concentrations of fat, carbohydrates, sodium, and protein [3]. The long term consumption of a high-fat (HF) diet can lead to various medical conditions, including hyperlipidemia, diabetes, and cardiovascular disease [4]. The overall goal of this study was to utilize both global and targeted liquid chromatography mass spectrometry (LC-MS) to assess the metabolic changes in rats maintained on a HF diet based on genetic predisposition (rat strain), diet, and age.

Non-alcoholic fatty liver disease (NAFLD) is one of the most common chronic liver disorders in the world [5]. Various HF diets have been used in different rat strains to assess the development and progression of NAFLD as characterized by hepatic steatosis, elevated triglyceride levels, changes in alanine transaminase (ALT), and a spectrum of histopathological changes in the liver (as reviewed by Kucera and Cervinokova, 2014; Van Herck et al., 2017) [5, 6]. Additionally, the effect of age has been shown to influence metabolism in a rat model [7]. After food intake, the ability to process glucose is significantly different over the lifespan of an animal. Ghezzi et al. reported that increased age caused changes in the metabolism of albino Wistar rats [7]. These changes included increased serum lipids, triglycerides, and glucose [7]. The age effect on the metabolism of the Wistar rats showed that glucose utilization and lipid metabolism efficiency changed over the lifespan of the animals and is an important variable to consider when planning a metabolism study.

Modern analytical techniques, including metabolomics, allow for a better understanding of the biomolecules and pathways involved in diet-induced disease states [8]. The metabolome is characterized by the full complement of small molecule metabolites found in the tissue, bio-fluids, and cells of an organism. These metabolites consist of a wide range of chemical classes including, but not limited to lipids, amino acids, monosaccharides, fatty acids, peptides, and nucleotides [9]. High resolution accurate mass (HRAM) mass spectrometry, coupled to ultra-high-performance liquid chromatography, can provide a rapid, accurate and quantitative profile of the metabolome. Liquid chromatography mass spectrometry was chosen over nuclear magnetic resonance (NMR) and gas chromatography mass spectrometry (GC-MS) due to the increased sensitivity at lower sample volumes and the ability to detect and quantify metabolites without the need for derivatization. Exploring the phenotypes associated with disease states, such as obesity, may lead to a greater understanding of the processes that cause the disease [10].

In this study we used liquid chromatography mass spectrometry (LC-MS) to examine the global metabolic changes caused by the maintenance of a HF diet on three distinct strains of rat. Two inbred strains of rat [Brown Norway (BN) and Fisher 344 (F344)] and one outbred strain [Sprague Dawley (SD)] were used to examine the genetic influence of diet on the metabolome. Three time points were chosen to assess the effect of age on the metabolome of the different strains and diets. The results of this study will provide valuable insight into the selection of diet, rat strain, and age for future metabolic studies.

## Methods

### Animals and diet

Male Sprague-Dawley and Brown Norway rats were acquired from Hilltop Lab Animals, Scottdale, PA, USA. Male Brown-Norway rats were acquired from Harlan Laboratories, Inc., Indianapolis, IN, USA. The rats were received at 5 weeks of age and were free of pathogens and parasites. The rats from each strain were acclimated for 5 days upon arrival and were provided tap water and irradiated Teklad 2918 standard 18% protein rodent diet (Envigo Teklad Diets, Madison, WI, USA) *ad libitum*. The nutritional composition of the Teklad 2918 regular diet was 18.6% protein, 44.2% carbohydrate, and 6.2% fat.

Post acclimation, a set of animals from all three strains were continued Teklad 2918 diet (N = 48 rats/strain) or started on the Teklad Custom 45% HF Kcal, Western Diet (Envigo Teklad Diets, Madison WI, USA; n = 48/strain) *ad libitum*. The 45% HF Kcal Western Diet was supplemented with 21% anhydrous milk fat and 34% sucrose. In order to supplement essential fatty acids, soybean (2%) was added to the HF diet. The nutritional composition of the HF diet was 14.8% protein, 40.6% carbohydrate, and 44.6% fat.

A subset of rats from each strain were maintained on the regular or HF diet until humanely euthanized at 4, 12, and 24 weeks by an intraperitoneal injection of sodium pentobarbital euthanasia solution (>100 mg/kg body weight; Fatal-Plus Solution, Vortech Pharmaceutical, Inc., Dearborn, MI) followed by exsanguination of the abdominal aorta. All animal procedures used during the study were reviewed and approved by the CDC-Morgantown Institutional Animal Care and Use Committee. All animal facilities used in this study were specific pathogen-free, environmentally controlled, and accredited by the AAALAC, International (Frederick, MD).

### Serum triglyceride measurement

Serum was collected from whole blood at the 4, 12, and 24-week timepoints. The serum was then analyzed using a Catalyst DX Bioanalyzer from IDEXX (North Grafton, MA). A total of 300 μL of rat serum was analyzed using the rodent triglyceride slide from IDEXX. There was no dilution performed prior to analysis.

### Statistical analysis for serum triglycerides

Analysis of variance (ANOVA) of serum triglycerides was performed using JMP ver. 12. In order to meet the assumptions for ANOVA and reduce heterogenous variance, the data was log transformed. The threshold for significance was set at p<0.05.

### Liver sample preparation

Excised livers were placed into 2 mL cryogenic vials and stored at -80°C until sample preparation and analysis. A 100 mg piece of each liver was removed and placed into 2 mL Eppendorf tube along with ~100 zirconia beads (1.5 mm dia.). Tissues were homogenized through bead beating at maximum speed for 2 minutes at 4°C in 250 μL of an extraction buffer of ice cold 80% methanol/20% water. Homogenized samples were then centrifuged at 14,000 x G for 10 minutes at 4°C to pellet proteins and cellular debris. The supernatants were then removed and dried using a Speedvac. The dried metabolite extracts were reconstituted in 50 μL of 50% methanol/water and used for LC-MS analysis. A portion of each liver was also kept for Oil Red O staining for histopathological analysis of lipid changes.

### Analytical procedure and LC-MS conditions for global metabolomics

The metabolite extraction was performed with a modified version of a previously described method by Boyce et al. [11]. In brief, the Agilent LC-MS system (Agilent Technologies, Santa Clara, CA, USA) included an Agilent 1290 Infinity quaternary ultra-high-pressure liquid chromatography (UHPLC) pump. This LC system was coupled to an Agilent 6530 quadrupole time of flight (Q-ToF) mass spectrometer with electrospray ionization. The chromatographic separations were performed using a Merck polymeric bead based ZIC-pHILIC column (100mmx2.1mm, 5μm).

The mobile phase A consisted of 10 mM ammonium acetate that was maintained at pH 8.5, and mobile phase B consisted of 10 mM ammonium acetate in 100% acetonitrile that was also maintained at pH 8.5. The separation gradient went from 95% mobile phase A to 5% mobile phase B over 20 minutes with a 7-minute re-equilibration at the end of each chromatographic run.

The mass spectrometer was operated in both positive and negative ion mode for the analysis. There were two technical replicates acquired for each sample. The MS/MS analysis was performed using identical chromatography conditions in order to confirm metabolite identity. A fixed collision energy of 30 kV was used for the data dependent MS/MS analysis.

### Data processing and metabolite identification for global metabolomics

Data processing was performed using a method described by Boyce et al. [11]. In brief, global LC-MS metabolomic analysis was performed on the extracted liver metabolites. The LC-MS/MS data was acquired using Agilent Mass Hunter Workstation (*.d files) and processed in Agilent Profinder software version 2.3.1 for batch recursive analysis. The datasets were subjected to spectral peak extraction with a minimum peak height of 1000 counts, and the charge state for each metabolite was restricted to two. Retention time and mass alignment was performed using Agilent Profinder software version 2.3.1. The resulting features then were exported as *.cef files to Mass Profiler Professional (MPP) software version 2.4.3 (Agilent Technologies, Santa Clara, CA, USA) for statistical analysis and metabolite identification through the embedded METLIN metabolite database. Metabolites were putatively identified based on accurate mass (<10 ppm mass error) and then confirmed through MS/MS fragmentation and spectral library searches.

### Statistical data analysis for metabolomics

Mass Profiler Professional software, from Agilent, was used for statistical analysis. One-way ANOVA was performed to obtain differences between experimental groups. Only the analytes with p values < 0.05 and fold change (FC) > 2 were treated as statistically significant. Bonferroni test corrections were applied to reduce false positives and false negatives in the data. A principal component analysis was performed for pattern recognition within the data using Mass Profiler Professional. Pareto scaling was utilized on the data prior to performing the PCA.

### Targeted metabolomics

The absolute quantification of the TCA cycle intermediates was performed on the liver metabolite extracts using a 5500 QTRAP mass spectrometer (SCIEX Inc., Concord, Ontario, Canada). Chromatographic separations were performed using an HPLC system consisting of two Shimadzu LC 20 AD pumps that included a Shimadzu SIL 20 AC auto sampler (Shimadzu Corp. Kyoto, Kyoto Prefecture, Japan). Metabolites were separated on a 3.0x150 mm Imtakt Scherzo SW-C18 column (Imtakt USA, Portland, Oregon). The column oven temperature was maintained at 40°C for the analysis. The analysis included a 5 μL injection volume for each sample. Mobile phase A consisted of 10 mM ammonium acetate with 0.3% formic acid, and mobile phase B was 100% acetonitrile with 0.3% formic acid. The chromatography gradient began at 5% B for 0.5 minutes and was increased to 95% B over 10 minutes followed by re-equilibration to initial conditions for 2.5 minutes. The flow rate for the analysis was 0.3 mL/minute.

Multiple reaction monitoring was used for the quantification of targeted metabolites. Mass spectrometer source conditions were as follows: curtain gas = 20, spray voltage = -4800 V, source temperature = 600°C, source gas 1 = -45, source gas 2 = -50. Calibration concentrations ranged from 0.05 to 10 ng/mL. In order to assess variation in the assay duplicate standard curves were analyzed at the beginning and at the end of the analytical run. Absolute quantification data was processed using Analyst software ver. 1.6.3 (SCIEX Inc., Concord, Ontario, Canada).

### Chemicals and reagents

Ultra-pure Optima Acetonitrile, methanol, and LC-MS grade Water were acquired from Thermo Fisher Scientific (Thermo Fisher Scientific, San Jose, CA). Analytical standards for succinic acid >99.5% purity, lactic acid >99% pure, citric acid >99% pure, malic acid >99% pure, pyruvic acid > 99% pure, α-ketoglutaric acid >95% pure, fumaric acid >99% pure, and an internal standard malic acid-d3 were acquired from TRC (Toronto Research Chemical, Toronto, Canada).

## Results

### High fat diet

**Fig 1:**
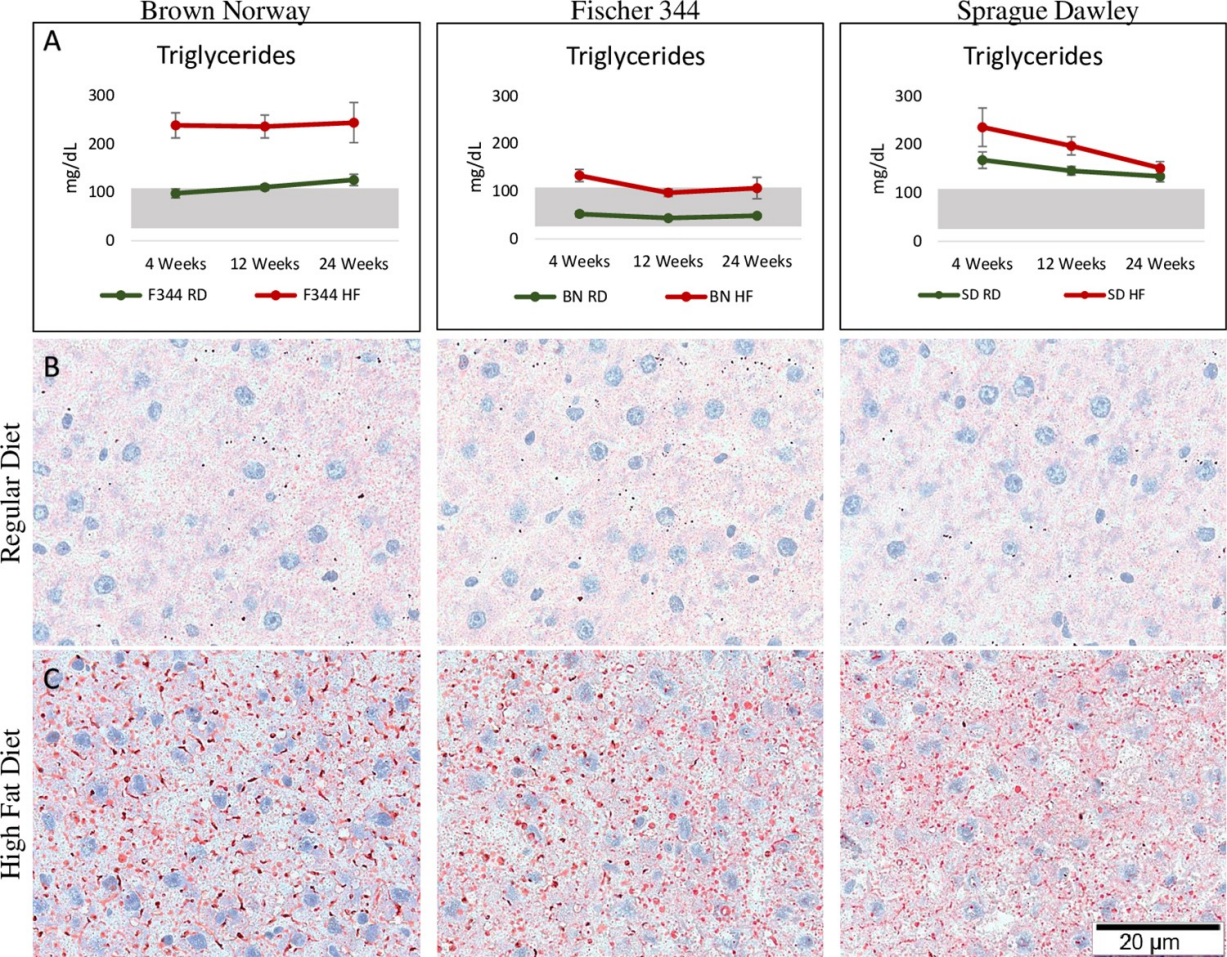
A) Serum triglyceride levels from all three strains over the 24-week time course. B) Liver sections from regular diet fed animals at the 12-week time point stained with Oil O Red at 20X magnification. C) High fat diet fed animal liver sections at the 12-week time point stained with Oil O Red at 100X magnification showing lipid changes in the tissue.

The animals maintained on the HF diet from all three strains had significantly higher levels of serum triglycerides at most time points compared to the group of animals fed the regular diet (Fig 1A). Animals fed the HF diet from each strain mostly demonstrated serum triglyceride levels higher than the normal range (>110 mg/dL), specifically for the BN and SD strains. The animals from the inbred strains, BN and F344, maintained on the regular diet had serum triglyceride levels within normal range (40–110 mg/dL), whereas the outbred SD strain had levels above the normal range, even when fed a regular diet. Along with elevated levels of serum triglycerides, the animal maintained on the HF diet had evidence of steatosis during the 24-week feeding regimen. Representative images of fresh frozen liver sections stained with Oil Red O showing a substantial increase in lipid accumulation (areas stained red) in the liver tissue of the animals fed the HF diet for each strain compared to the regular diet controls at 12 weeks for each strain (Fig 1B and 1C).

### Global metabolomics

**Fig 2:**
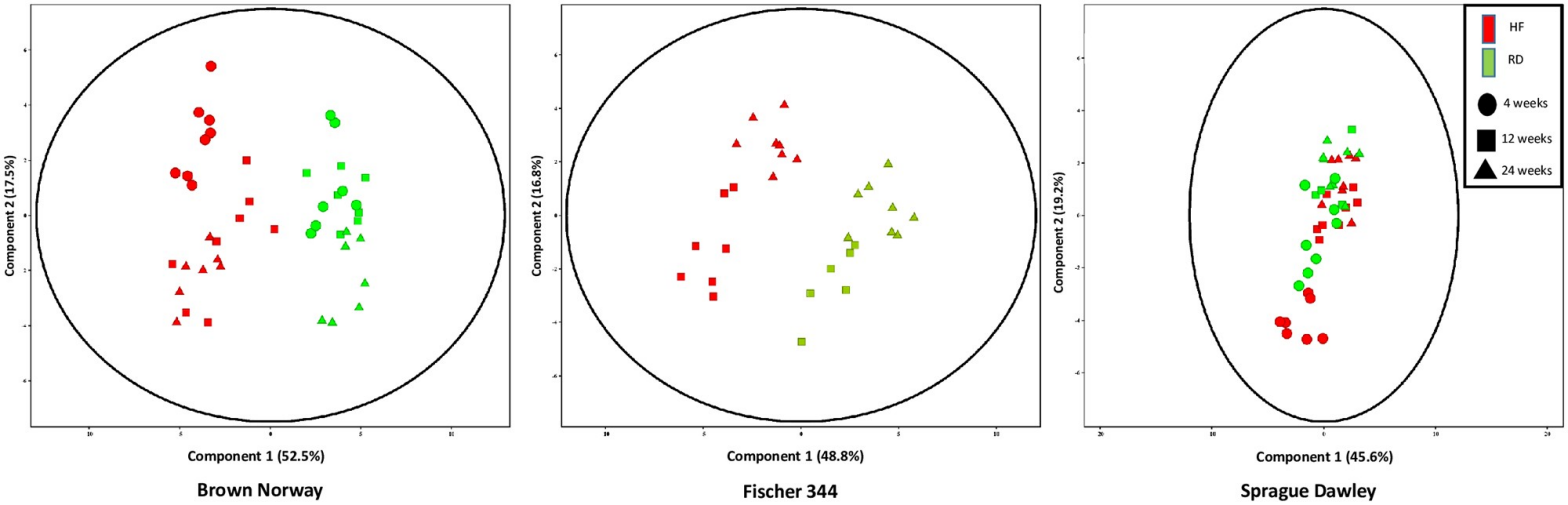
PCA scores plots obtained from UHPLC-Q ToF-MS data of the global liver metabolome from three rat strains fed either high fat (HF) or regular diet (RD) and at three different time points (4 weeks, 12 weeks, and 24 weeks). The area inside the ellipse region represents the 95% confidence interval.

A total of 1243 unique chromatographic features were detected from the liver global metabolomics analysis. The global metabolome of all three strains was influenced by both diet and age with diet causing the greater variation of the two factors (Fig 2). Although all three strains were influenced by both age and diet, the SD strain showed the least pronounced separation in the components. The BN and F344 strains were metabolically influenced more by diet than age, demonstrating a very distinct separation between the HF and regular diet groups. The 4-week time point is missing for the Fisher 344 strain due to a loss of sample prior to analysis.

When comparing the total number of significantly changing metabolites between all three strains, the number was greater based on diet than age. The total effect of the diet on all three strains combined yielded 28 total metabolites significantly changing that were identified through MS/MS fragmentation library searching (Table 1). Of the 28 significantly changing metabolites, five were downregulated in the HF diet group compared to the regular diet group. These reduced metabolites that were shared amongst all three strains included (20:4) Lyso-phosphatidylcholine, 2-hydroxydodecanimidic acid, serotonin, and 18:1 (Omega-9) Lyso-phosphatidylcholine. These metabolites are primarily involved in the lipid metabolism pathway and insulin signaling. The 23 upregulated metabolites that were observed in the HF diet group were primarily involved in carnitine and amino acid metabolism. The carnitine metabolism pathway intermediates that were increased in the HF diet animals included g-Butyrobetaine, L-Palmitoylcarnitine, O-oleoylcarnitine, L-Carnitine, and L-Lysine.

**Table 1:** Significantly changing metabolites between the high fat diet and regular diet for all three strains. Metabolites identified and confirmed via MS/MS fragmentation and spectral library searching.

Compund Name	Molecular Weight	RT [min]	Log2 Fold Change:HF/RD Brown Norway	Log2 Fold Change:HF/RD Fischer 344	Log2 Fold Change:HF/RD Sprauge Dawley	Metabolic Pathway	
LysoPC (20:4)	543.33379	4.508	-4.85	-2.85	-3.05	Lipid Metabolism	HMDB0010395
13-KODE	294.21993	1.36	-2.15	-2.34	-2.32	Lipid Metabolism	HMDB0004668
Serotonin	176.13091	8.745	-2.83	-2.36	-2.13		HMDB0000259
2-hydroxydodecanimidic acid	495.42894	12.898	-2.65	-2.1	-2.05	Insulin Signaling	HMDB0035480
2–18:1(Omega-9) LPC	521.6761	4.979	-1.13	-2.13	-1.13	Lipid Metabolism	HMDB0000378
2-methylbutyrylcarnitine	245.16319	1.901	-1.59	1.86	1.26	Lipid Metabolism	HMDB0000378
Eicosapentanoic acid	302.22469	1.328	1.95	1.65	1.34	Lipid Metabolism	HMDB0001999
Indoleacrylic acid	187.06339	6.277	2.31	1.75	1.54		
g-Butyrobetaine	145.11027	2.661	1.85	1.85	1.73	Carnitine Synthesis	HMDB0001161
L-Palmitoylcarnitine	399.33583	1.71	1.65	2.15	1.97	Carnitine Synthesis	HMDB0000222
L-(-)-Threonine	119.05845	7.264	2.26	2.19	2.13	Glycine and Serine Metabolism	HMDB0000167
O-oleoylcarnitine	425.35052	1.355	2.36	2.38	2.26	Carnitine Synthesis	HMDB0005065
Glycerol	428.31261	1.285	2.42	2.57	2.49	Lipid Metabolism	HMDB0000131
3-Hydroxytetradecanedioic acid	274.17796	1.339	2.85	2.89	2.7	Lipid Metabolism	HMDB0000394
DL-Carnitine	161.10573	5.439	2.95	2.95	2.8	Carnitine Synthesis	HMDB0000062
Dihydrothymine	128.05868	7.47	3.24	3.25	3.07	Pyrimidine Metabolism	HMDB0000079
carnosine	226.10645	9.06	4.5	3.78	3.17	Beta-alanine Metabolism	HMDB0000033
L-Pyroglutamic acid	129.04279	7.461	2.84	2.95	3.34	Glutathione Metabolism	HMDB0000267
Acetylcarnitine	203.11603	2.729	2.66	3.21	3.43	Lipid Metabolism	HMDB0000201
Fructoselysine	308.15839	9.508	3.58	4.15	3.49		HMDB0034879
DL-Lysine	146.10548	9.052	4.26	4.35	4.1	Carnitine Synthesis	HMDB0000182
2’-Deoxycytidine	227.09044	3.095	4.48	4.68	4.43	Pyrimidine Metabolism	HMDB0000014
Homocarnosine	240.12215	9.003	4.98	5.25	5.72		
DL-Arginine	174.11166	8.859	5.85	6.85	6.25	Glycine and Serine Metabolism	HMDB0000517
DL-Histidine	155.06946	8.703	6.3	7.12	6.41	Histidine Metabolism	HMDB0000177
Acetyl-CoA	809.1258	2.6	2.3	1.5	3.6	Lipid Metabolism/TCA Cycle	HMDB0001206
Malate	132.0058	5.26	0.89	0.12	-0.46	TCA Cycle	HMDB0000156
Fumarate	114.057	4.25	1.89	1.65	0.25	TCA Cycle	HMDB0000134
Succinate	116.0109	3.27	-0.98	-1.12	-0.54	TCA Cycle	HMDB0000254
Homo-L-arginine	188.12726	8.729	7.25	7.68	7.65		HMDB0000670

*All identified metabolites have a p value ≤0.05.

The global metabolomics also showed changes in intermediates of the tricarboxylic acid cycle (TCA), including acetyl CoA, malate, succinate, and fumarate. These intermediates were changed by both diet and age for all three strains. These relative quantitative changes in the TCA cycle intermediates were further analyzed using a targeted absolute quantification.

### Targeted metabolomics

**Fig 3:**
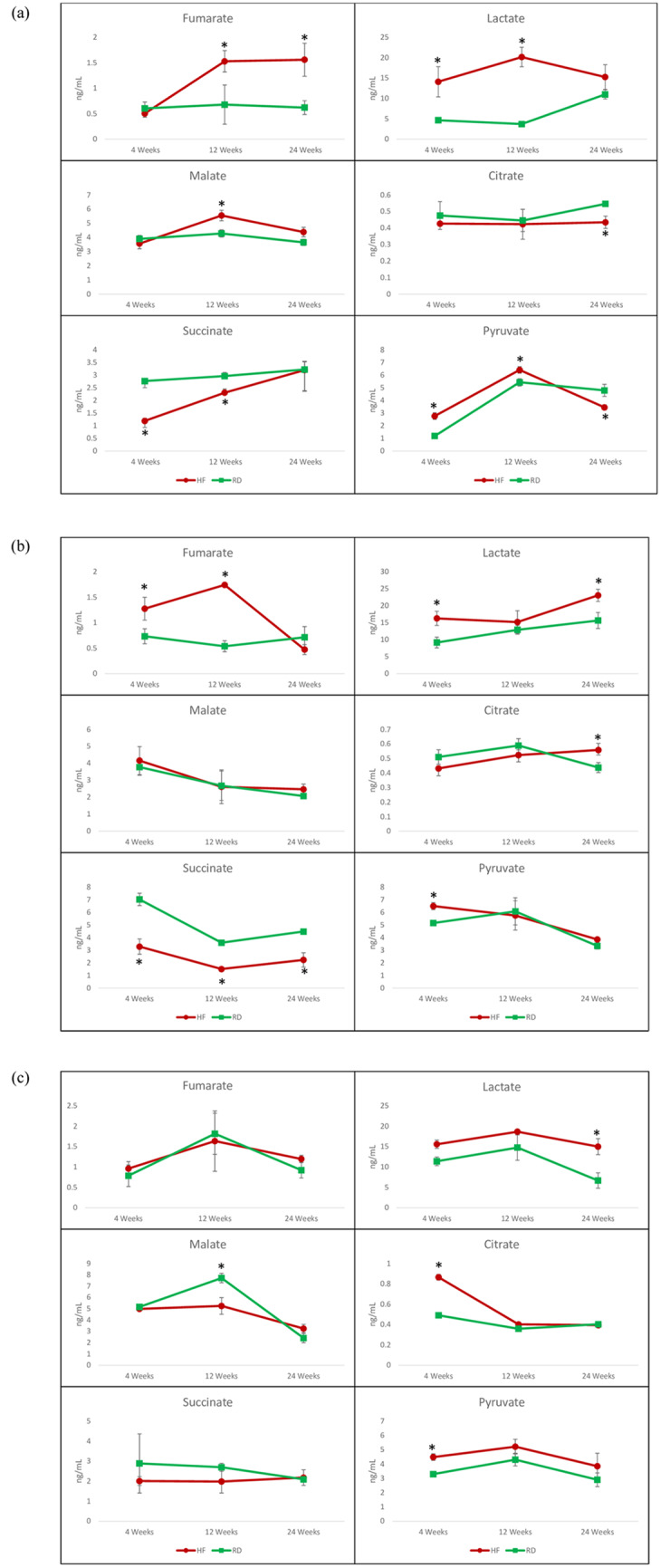
A) Concentrations of liver tricarboxylic acid cycle intermediates for the Brown Norway Strain high fat diet (HF) and regular diet (RD). B) Concentrations of liver tricarboxylic acid cycle intermediates for the Fischer 344 high fat diet (HF) and regular diet (RD). C) Concentrations of liver tricarboxylic acid cycle intermediates for the Sprague Dawley Strain high fat diet (HF) and regular diet (RD). * p = <0.05.

Citrate, succinate, fumarate, pyruvate, malate, and lactate were quantified from liver extracts from each sample. The targeted quantification analysis revealed that all three strains had significant changes in the TCA cycle intermediate concentrations from liver tissue analysis based on diet. The concentration of citrate in the liver tissue samples of the SD strain for the regular diet was 0.491 (±0.025) ng/mL compared to 0.862 (±0.025) ng/mL for the SD strain maintained on the HF diet at 4 weeks (Fig 3C). This was the highest observed concentration of citrate for all three strains. The two inbred strains, F344 and BN, had no significant changes in the citrate concentrations at the 4 and 12-week timepoints, however the concentration of citrate was significantly higher at the 24-week timepoint for the high fat diet fed F344 and significantly lower in BN strain. The F344 strain exhibited a significant increase of fumarate and pyruvate at the 4-week time point in the HF diet-fed groups compared to the regular diet, while the SD strain had no significant difference in fumarate at the 4-week timepoint. Succinate concentrations were found to be significantly higher at the 4 and 12-week timepoints in the two inbred strains, while there was significant change in succinate concentrations in the outbred SD strain. In contrast to succinate, the lactate concentrations were significantly higher in the HF diet fed inbred stains (Fig 3A and 3B) and significantly higher only at the 24-week timepoint for the outbred SD strain (Fig 3C). The changes observed in the liver TCA cycle were most pronounced at the 4 and 12-week time points and were followed by a stabilization at the 24-week time point for the outbred SD strain, while the two inbred strains still showed significant changes at the 24-week time point.

## Discussion

In the present study, we monitored global and targeted hepatic metabolite changes in the liver of three genetically distinct rat strains that were maintained on both a regular and high fat diet over a period of 24 weeks. LCMS was used to generate metabolic profiles, and the responses were compared using multivariate statistics to identify significantly altered metabolites for each strain over the evaluation period. Interestingly, diet had a greater influence on the metabolic changes of the two inbred strains in contrast to age being a greater influence on the outbred Sprague Dawley strain.

The HF diet was associated with elevated serum triglycerides observed steatosis was ubiquitous across each strain and time point for the HF diet-fed animals, a finding confirmed in other similar studies comparing steatosis in different rat strains [12, 13].

Lipid metabolism and carnitine biosynthesis, along with changes in the TCA cycle intermediates, were the metabolic pathways found to be most significantly affected across all three strains. The diet-induced changes in hepatic metabolism were in agreement to those reported in previous studies [14–16]. Changes in the TCA cycle enzyme activity in HF diet-fed mice had been shown by Satapi et al. [17]. This study showed that intermediates from the TCA cycle were being rerouted to gluconeogenesis through changes in enzyme activity when mice were fed a HF diet. The potential rerouting of these metabolic intermediates to gluconeogenesis in hepatic tissue is a potential indicator of diet-induced insulin resistance [18]. This observed concentrations in TCA cycle intermediates in mice fed a HF diet correlated with the increase levels of fumarate in the inbred rat strains and citrate in the outbred SD strain fed a HF diet. The disruption in the levels of these TCA cycle intermediates could induce anaplerotic reactions that are required to replenish these intermediates that are being rerouted to gluconeogenesis.

The presence of steatosis in the liver has been shown to be the primary indicator of NAFLD and hepatic insulin resistance [19]. Metabolites associated with NAFLD were upregulated in the animal strains maintained on the HF diet, including lactate in the outbred Sprague Dawley strain and lactate and fumarate in the two inbred strains, which are involved in both amino acid metabolism as well as the TCA cycle. The elevated levels of these two metabolites have been linked to hepatic fibrosis associated with NAFLD [20]. The inbred BN and F344 strains of rats maintained on the HF diet showed relatively consistent higher levels of both lactate and fumarate, whereas it was less pronounced in the outbred SD strain, which had significantly higher levels of lactate at only 4 and 24 weeks.

A reduction of hepatic metabolites involved in β-oxidation and lipid metabolism, as well as the changes in the metabolites involved in the carnitine shuttle could explain the development of steatosis. The carnitine shuttle is an important pathway in all animals and is responsible for moving long chain fatty acids across the mitochondrial membrane where they will undergo β-oxidation [21]. The acetyl-CoA product of β-oxidation is then fed into the TCA cycle [22]. The disruption of these metabolic processes due the consumption of a HF diet could lead to a buildup of unprocessed lipids in the hepatic tissue causing the observed steatosis in all three strains. Further investigation is ongoing to confirm this observation.

Interestingly, liver serotonin was found to be ~2.0 fold lower in the HF diet-fed animals compared to the rats maintained on the regular diet for all three strains. The results of a previous study that showed increased serum serotonin levels in obese mice, but there is little data on the hepatic serotonin levels of HF diet fed rats [23]. Serotonin has been shown to regulate hepatic lipid metabolism and lipid homeostasis [24]. However, the dysregulation of hepatic serotonin could play a role in appetite control in the different rat strains, leading to increased food intake on a high fat diet.

## Conclusions

The results of this study show that diet, strain, and age can play a significant role in the hepatic metabolism of rats. The influence of diet on all three rat strains used in this study primarily changed the lipid metabolism pathway. There were also observed changes in the carnitine pathway, as well as amino acid metabolism. The most significant changes were observed in the targeted quantification of TCA cycle intermediates from the livers of all three strains. The selected inbred strains (BN and F344) were much more sensitive to the HF diet than the outbred strain (SD). While all three strains showed that age effected their metabolism the inbred strains global metabolome showed greater sensitivity than the outbred strain. The use of high-resolution accurate mass spectrometry combined with ultra-high performance liquid chromatography allowed for the sensitive and accurate quantification of these metabolites from the hepatic tissue. These observations are important for selecting a specific rat strain for a metabolic study. Understanding the differences in metabolic capacities of these three strains will help guide the choice of these animals for future studies.

## Supporting information

S1 Fig A) Representative chromatogram from a Fisher 344 liver metabolite extract in positive ion mode acquired on a Q-ToF mass spectrometer. B) Total ion current spectra from the chromatographic run in A).
(PPTX)

S1 Table MS/MS parameters for each TCA cycle intermediate analyzed on a Qtrap MS. (PPTX)

S1 Data (XLSX)

S2 Data (XLSX)
