# Supplementary material for: Using liquid chromatography mass spectrometry (LC-MS) to assess the effect of age, high-fat diet, and rat strain on the liver metabolome
Source: PLoS One. 2020 Jul 1;15(7):e0235338. doi: 10.1371/journal.pone.0235338 (PMC7329071; doi:10.1371/journal.pone.0235338)
Supplement: S1 Fig — A) Representative chromatogram from a Fisher 344 liver metabolite extract in positive ion mode acquired on a Q-ToF mass spectrometer. B) Total ion current spectra from the chromatographic run in A). (PPTX) [file pone.0235338.s001.pptx]

## Slide 1
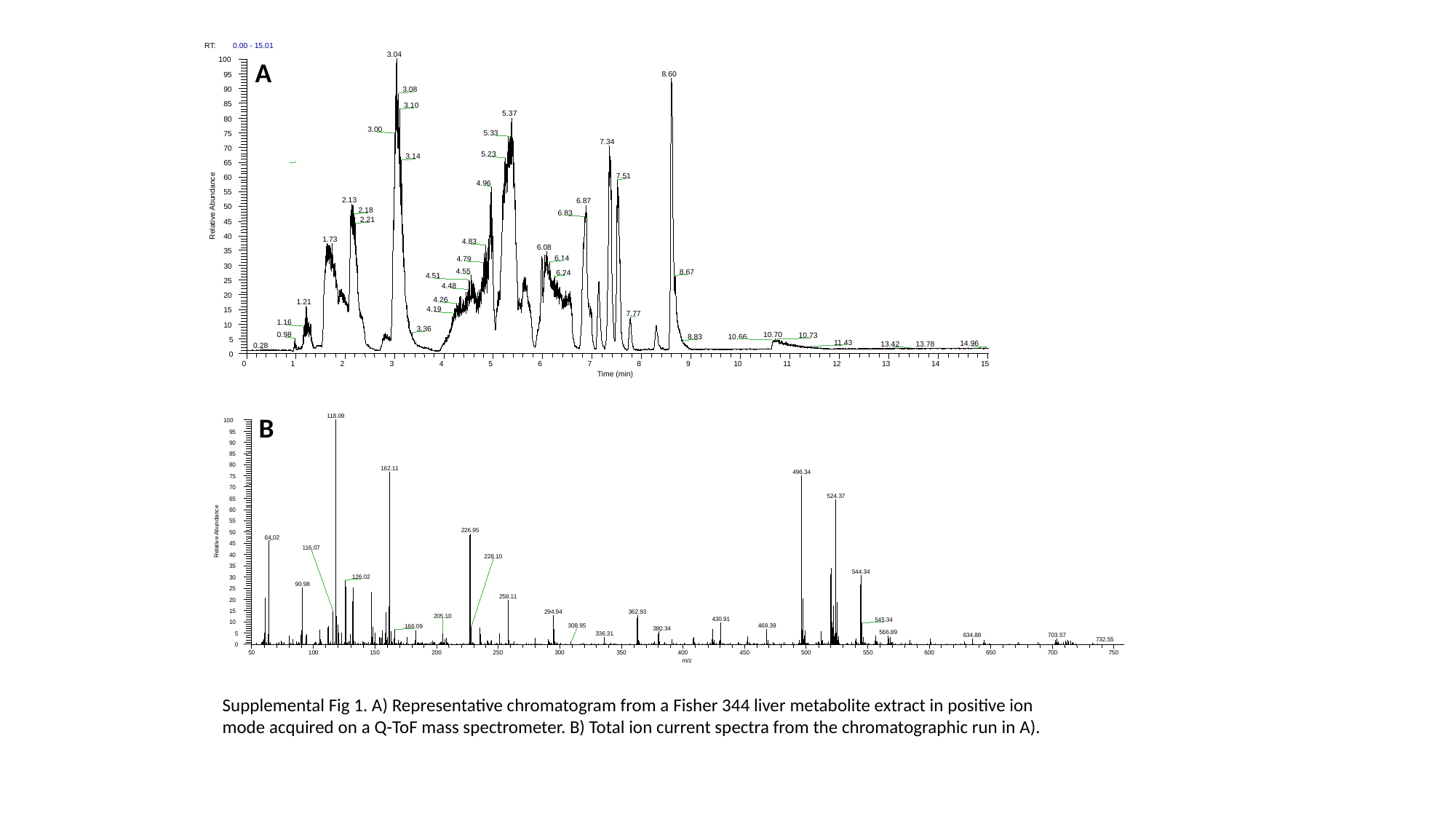

RT:
0.00 - 15.01
50
45
40
35
30
25
20
15
10
5
0
0
1
2
3
4
5
6
7
8
9
10
11
12
13
14
15
Time (min)
3.04
100
8.60
95
90
3.08
85
3.10
5.37
80
3.00
5.33
75
7.34
70
5.23
3.14
65
7.51
60
4.96
55
2.13
6.87
Relative Abundance
2.18
6.83
2.21
1.73
4.83
6.08
6.14
4.79
4.55
8.67
6.24
4.51
4.48
4.26
1.21
4.19
7.77
1.16
3.36
10.70
0.98
10.73
10.66
8.83
11.43
14.96
13.42
13.78
0.28
40
35
30
25
20
15
10
5
0
50
100
150
200
250
300
350
400
450
500
550
600
650
700
750
m/z
118.09
100
95
90
85
80
162.11
496.34
75
70
524.37
65
60
55
226.95
Relative Abundance
50
64.02
45
116.07
228.10
544.34
126.02
90.98
258.11
294.94
362.93
205.10
430.91
545.34
308.95
468.39
166.09
380.34
566.89
336.31
634.88
703.57
732.55
A
B
Supplemental Fig 1. A) Representative chromatogram from a Fisher 344 liver metabolite extract in positive ion mode acquired on a Q-ToF mass spectrometer. B) Total ion current spectra from the chromatographic run in A).
