# Supplementary material for: Using liquid chromatography mass spectrometry (LC-MS) to assess the effect of age, high-fat diet, and rat strain on the liver metabolome
Source: PLoS One. 2020 Jul 1;15(7):e0235338. doi: 10.1371/journal.pone.0235338 (PMC7329071; doi:10.1371/journal.pone.0235338)
Supplement: S1 Table — (PPTX) [file pone.0235338.s002.pptx]

## Slide 1
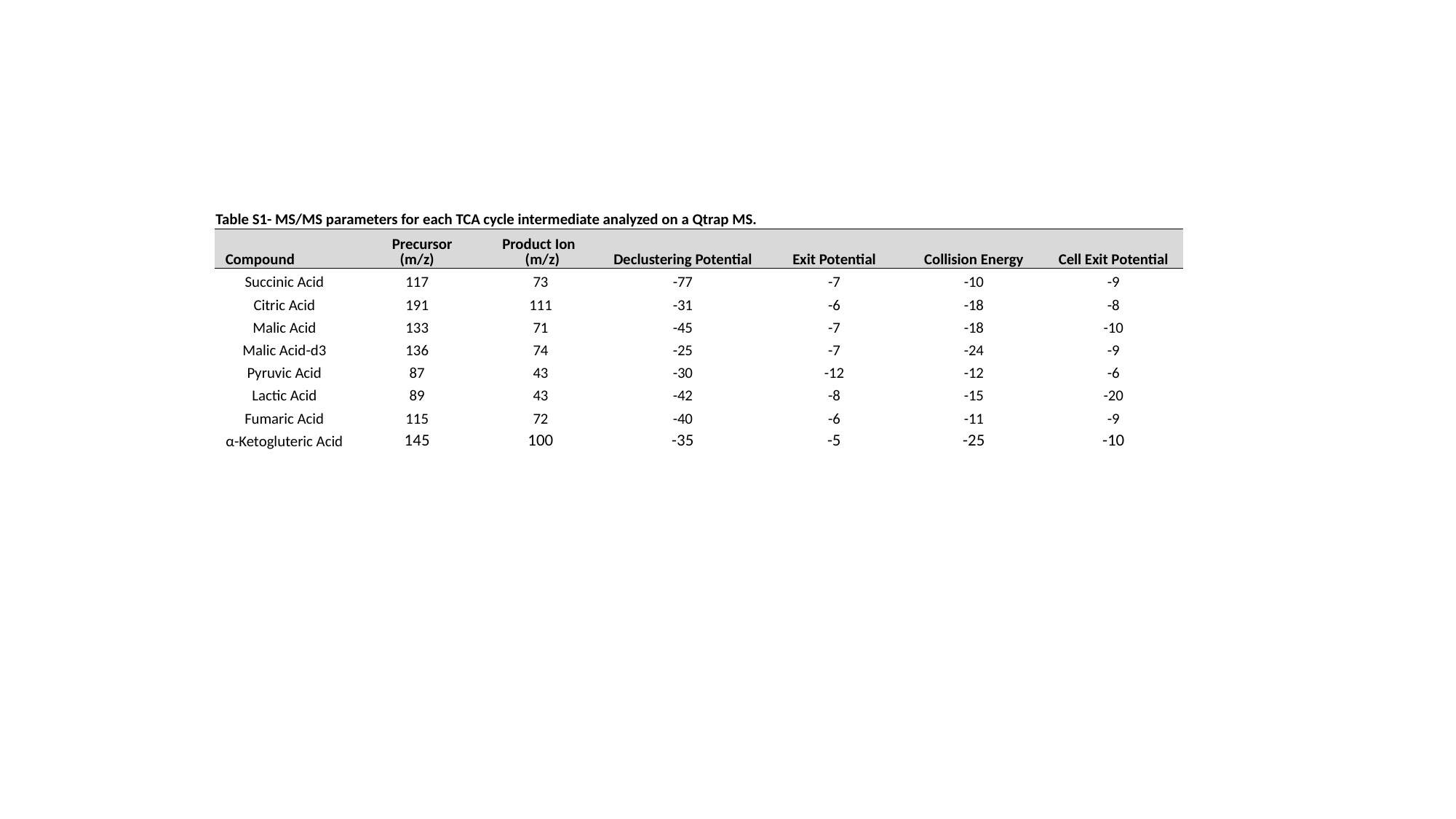

| Table S1- MS/MS parameters for each TCA cycle intermediate analyzed on a Qtrap MS. | | | | | | |
| --- | --- | --- | --- | --- | --- | --- |
| Compound | Precursor (m/z) | Product Ion (m/z) | Declustering Potential | Exit Potential | Collision Energy | Cell Exit Potential |
| Succinic Acid | 117 | 73 | -77 | -7 | -10 | -9 |
| Citric Acid | 191 | 111 | -31 | -6 | -18 | -8 |
| Malic Acid | 133 | 71 | -45 | -7 | -18 | -10 |
| Malic Acid-d3 | 136 | 74 | -25 | -7 | -24 | -9 |
| Pyruvic Acid | 87 | 43 | -30 | -12 | -12 | -6 |
| Lactic Acid | 89 | 43 | -42 | -8 | -15 | -20 |
| Fumaric Acid | 115 | 72 | -40 | -6 | -11 | -9 |
| α-Ketogluteric Acid | 145 | 100 | -35 | -5 | -25 | -10 |
